# Supplementary material for: Development of chemokine network inhibitors using combinatorial saturation mutagenesis
Source: Commun Biol. 2025 Apr 3;8:549. doi: 10.1038/s42003-025-07778-6 (PMC11969024; doi:10.1038/s42003-025-07778-6)
Supplement: Supplementary file 4 — Supplementary Data 2 [file 42003_2025_7778_MOESM4_ESM.zip › SupplementaryData3/scripts/xattune_dr.tar!/./script/analysis/JK/JK_E169_CCL7_THP1_plot.pdf]

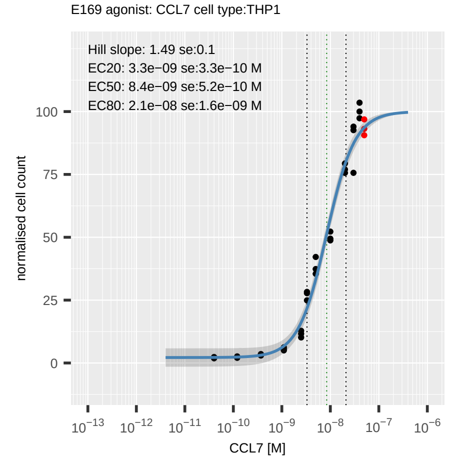
E169 agonist: CCL7 cell type:THP1
Hill slope: 1.49 se:0.1
EC20: 3.3e−09 se:3.3e−10 M
EC50: 8.4e−09 se:5.2e−10 M
EC80: 2.1e−08 se:1.6e−09 M
normalised cell count
0
25
50
75
100
10−13
10−12
10−11
10−10
10−9
10−8
10−7
10−6
CCL7 [M]
